# Transcriptomic Characterization of miRNAs in *Pyrrhalta aenescens* Fairmaire in Response to 20-Hydroxyecdysone Treatment

**DOI:** 10.3390/genes16040435

**Published:** 2025-04-05

**Authors:** Jie Liu, Li Gao, Chao Du, Tianfeng Duan, Li Liu

**Affiliations:** College of Ecology and Environment, Baotou Teachers’ College, Baotou 014030, China; tunriyaoyun@163.com (J.L.); gaoli8905@163.com (L.G.); ductub@163.com (C.D.); liuli4304842@126.com (L.L.)

**Keywords:** reproduction, hormone regulation, differentially expressed miRNAs (DEMs), functional enrichment, signaling pathway

## Abstract

Background/Objectives: *Pyrrhalta aenescens*, a major pest of elm trees, causes extensive ecological and economic damage through rapid population growth and defoliation. Existing research mainly focuses on its biological traits and chemical control, with little knowledge about its reproductive development mechanisms, a key factor in population expansion. In other insects, the steroid hormone 20-hydroxyecdysone (20E) regulates development and reproduction via microRNA (miRNA)-mediated pathways, but this has not been studied in *P. aenescens*. This study aimed to systematically identify miRNAs responsive to 20E in *P. aenescens* and unravel their roles in regulating reproduction and metabolic pathways, providing foundational insights into hormone–miRNA crosstalk in this ecologically significant pest. Methods: Adult beetles (collected from Baotou, Inner Mongolia) were injected with 1.0 μg/μL 20E or control. Total RNA from three biological replicates (10 adults each) was sequenced, followed by miRNA identification, differential expression analysis, target prediction, and functional enrichment. Results: Small RNA sequencing identified 205 miRNAs (162 conserved, 43 novel), with 12 DEMs post-20E treatment. Target prediction linked these miRNAs to 7270 genes, including key regulators of the *FoxO* signaling pathway and MAPK signaling pathway. KEGG analysis highlighted lipid metabolism and stress response pathways. Conclusions: This study revealed that 20E modulates miRNA networks to regulate *FoxO* and MAPK pathways in *P. aenescens*, suggesting hormonal control of lipid metabolism and developmental processes. As the first miRNA resource for this pest, our findings provide mechanistic insights into 20E–miRNA crosstalk and identify potential molecular targets for disrupting its reproductive biology, laying a foundation for eco-friendly pest control.

## 1. Introduction

*Pyrrhalta aenescens* (Coleoptera: Chrysomelidae) is a pest species that primarily harms plants in the Ulmus genus, including *Ulmus pumila* L., *U. pumila* L. ‘*Pendula*’, and *U. pumila* L. ‘Jinye’, such that it is a major pest in urban gardens. *P. aenescens* can be found throughout northern areas of China, including Heilongjiang, Shandong, and Inner Mongolia [1]. Both larvae and adults can damage the buds and leaves of elms, eating all of the leaves other than the veins in some instances [2]. According to statistics, a single elm tree suffering from infestation can experience a leaf loss rate exceeding 80%, directly resulting in diminished landscape value and an increased mortality rate of forest trees [3]. Given the high density of these insects, their singular feeding habits, and their fulminant characteristics, preventing and controlling *P. aenescens* infestations is challenging. In recent years, the damage caused by *P. aenescens* has been intensifying across northwestern China, northern China, and other regions, marked by expanding infestation areas, escalating severity, and gradually intensifying outbreak potential [4]. Therefore, the scientific prevention and control of *P. aenescens* have become particularly urgent and important. 

The generational cycles of these beetles vary based on local climatic conditions, which trigger the end of their overwintering hibernation. The adults then lay eggs in elm sprouts or proximal to the main veins of elm leaves. First-generation larvae emerge from May to June. Second-generation larvae emerge in early July and pupate in the cracks of tree trunks by early August. Following eclosion, they feed and then seek out suitable overwintering sites [5]. Current research on this pest has predominantly focused on its biological characteristics and chemical control [1,2,3,4,5,6], while lacking in-depth investigation into the core driver of population expansion—its reproductive development mechanisms. Therefore, elucidating the molecular regulatory networks governing its reproduction constitutes a critical breakthrough point for developing targeted control strategies.

The insect steroid hormone 20-hydroxyecdysone (20E), signaling through the ecdysone receptor (*EcR*) and ultraspiracle (USP), regulates physiological processes such as molting, metamorphosis, and reproduction by activating early-response genes (e.g., *E74*, *E75*, *E93* and *Br-C*) [7,8,9]. In this process, *E74* and *E75* act as heme sensors, regulating metamorphosis and reproduction in insects, while *Br-C* serves as a pupal stage signaling factor, functioning as a critical mediator during the larval–pupal transition [10,11,12]. Following the expression of these early-response factors, they induce the transcription of the early-late response factor *Ftz-f1*, which participates in the regulation of insect metamorphosis and reproduction [13]. Prior studies have sought to clarify the molecular mechanisms through which 20E functions. For instance, Hossain et al. [14] demonstrated the ability of 20E to enhance forkhead transcription factor (*FoxO*) activity and upregulate brummer and acid lipase-l expression, inducing fat body lipid degradation during the processes of molting and pupation in *Bombyx mori,* with 20E also regulating *Msr* expression via its effects on *FoxO* [15]. Huang et al. [16] further employed a high-throughput sequencing approach to explore how 20E affects fat body gene expression patterns in silkworm larvae. While 20E’s role in lipid metabolism and gene regulation has been explored in models like *B. mori*, its mechanisms in *P. aenescens* remain uncharacterized. This knowledge gap limits our ability to disrupt the pest’s life cycle effectively.

As small endogenous ~20 nucleotide noncoding RNAs, microRNAs (miRNAs) are ubiquitously expressed in eukaryotes, wherein they are encoded by endogenous genes and post-transcriptionally control gene expression through the inhibition of translation or the induction of mRNA degradation [17,18]. Past studies have suggested that miRNAs can influence insect reproduction, growth, and development by targeting key genes (e.g., *EcR*, *USP*, *Br-C*) in the ecdysone signaling pathway. Treating *Blattella germanica* with 20E, for instance, led to the upregulation of miR-1-3p and miR-100-5p, together with the downregulation of miR-252-3p [19], while miR-8 expression rose in *Drosophila* following 20E treatment [20]. Immediately following molting, silkworm Bmo-miR-14-5p and Bmo-miR-14-3p expression levels rose and suppressed ecdysone signaling-associated gene expression and 20E biosynthesis [21]. 

While these findings are informative, how most miRNAs interact with ecdysone signaling processes remains poorly understood in many species. With the exception of a genome-wide small RNA sequencing effort by Jin et al. [22] to characterize miRNA responses to 20E in embryonic cell lines from *B. mori* and *D. melanogaster*, all of these studies have only examined how 20E affects individual miRNAs. In *P. aenescens*, the molecular regulatory networks governing its reproduction, especially the role of 20E and its interaction with miRNAs, remain poorly understood.

High fecundity is a critical factor for insect survival, and insect reproduction is regulated by 20E. *P. aenescens*, a significant horticultural and forestry pest, has caused severe economic losses to these industries and related sectors. *P. aenescens* serves as an excellent model for studying insect reproduction, particularly in horticultural and forestry pests. Recent studies have demonstrated that miRNAs play pivotal regulatory roles in insect growth and development. Therefore, investigating the regulatory functions of miRNAs in the reproductive development of adult *P. aenescens* and exploring the stress response mechanisms of *P. aenescens* miRNAs to 20E will not only help elucidate the occurrence patterns and outbreak mechanisms of this pest—providing crucial theoretical and practical insights for its sustainable control—but also advance our understanding of the molecular mechanisms underlying reproductive development in forestry insects.

## 2. Materials and Methods

### 2.1. Insect Rearing

Larvae of *P. aenescens* were collected from elm trees in Baotou, Inner Mongolia, on 10 June 2023, and reared in the insect laboratory of Baotou Teachers College. Both larvae and adults were maintained in climate-controlled chambers at 25 ± 1 °C under a 16:8 h light–dark cycle, simulating natural summer photoperiods in northern China. Fresh *U. pumila* leaves were provided ad libitum daily, and humidity was maintained using moistened filter paper in rearing containers. Leaves and filter paper were replaced every 24 h to ensure freshness. Adults used in experiments were selected on day 3 post-eclosion. After injection with 20E or control solution, treated individuals were immediately returned to the original rearing conditions, ensuring a consistent temperature, humidity, photoperiod, and nutritional supply before and after treatment.

### 2.2. 20E Treatment and Sample Collection

Dimethyl sulfoxide (DMSO) was used to dilute 20E (CAS-number: 5289-74-7; purity: 99.64%; MedChemExpress, Shanghai, China) to 10 mg/mL, and this stock solution was stored at –80 °C. Working solutions of 20E were prepared by using normal saline to dilute the stock solution to a final concentration of 1.0 μg/μL, while DMSO diluted with normal saline served as the control solution. The abdomen of each adult used in this experiment was injected with 1 µL of the control or 1.0 μg/μL 20E treatment solutions on day 3 after eclosion using a microsyringe. The sampling of these individuals was performed 2 days post-injection [23,24,25]. Three biological replicates (10 adults each) were set up per treatment group, with each replicate sample individually ground in liquid nitrogen and stored at −80 °C prior to sRNA-Seq and qRT–PCR analyses.

### 2.3. RNA Sequencing

TRIzol Reagent (Invitrogen, Waltham, CA, USA) was used as directed to isolate total RNA from the whole body of adult specimens. The integrity and purity of this RNA was assessed using 1% agarose gels, while a NanoPhotometer^®^ spectrophotometer (IMPLEN, Westlake Village, CA, USA) and Qubit^®^ 2.0 Fluorometer (Life Technologies, Carlsbad, CA, USA) were used to assess RNA purity and concentration, respectively, and an Agilent Bioanalyzer 2100 (Agilent Technologies, Santa Clara, CA, USA) was used to measure RNA integrity. Small RNA libraries were prepared from 3 μg of RNA per sample, and library preparation and sequencing were performed as in a prior study published by Wang et al. [26]. The RNA sequencing was carried out by LC BioTechnology Co., Ltd. in Hangzhou, China, using an Illumina HiSeq 2500 instrument.

### 2.4. Small RNA Analyses and miRNA Identification

The ACGT101-miR (v4.2) software package (LC Sciences, Houston, TX, USA) was used for the processing of raw data by removing adapter dimers, junk sequences, low complexity reads, common RNA families, and repeat sequences. The remaining unique sequences 18–26 amino acids long were mapped to miRBase 22.1 via a BLAST search (v2.12.0+) to facilitate the identification of both novel and previously reported 3p- and 5p- derived miRNAs [27,28]. Variations in length at the 3′ and 5′ ends of a maximum of one mismatch within these sequences was permitted during alignment. The reference genome of adult *P. aenescens* was based on assembled transcriptomic data in response to 20E stimulation (GenBank No.: PRJNA1173923). Unmapped sequences were blasted against the *P. aenescens* transcriptome with BOWTIE, permitting a maximum of one mismatch [29]. Hairpin-containing RNA structure predictions were made based on flanking 80-nucleotide sequences in the RNA fold program (http://rna.tbi.univie.ac.at/cgi-bin/RNAWebSuite/RNAfold.cgi, accessed on 16 October 2023) [30]. Pre-miRNA sequence predictions were made through secondary structural analyses of mapped read sequences [31], with those sequences with stem-loop structures and flanking sequences found in the stem regions being classified as candidate *P. aenescens* miRNAs. Copy number correction across samples was achieved through modified global normalization [32].

### 2.5. Differential miRNA Expression Analyses

Transcripts per million (TPM) values were used to assess the expression of miRNAs as follows: Normalized expression = mapped (readcount/total reads) × 1,000,000 [33], and miRNAs with TPM ≥ 1 were retained for downstream analyses to exclude low-expression noise. Following treatment with 20E, differentially expressed miRNAs (DEMs) were identified with the R DESeq package (v 1.8.3) [34], following the “rlog” transformation to stabilize variance across samples. A negative binomial generalized linear model (GLM) was applied to compare the 20E-treated and control groups. Contrast comparisons were defined using the Wald test. Contrast comparisons were specified as ~treatment + batch to account for batch effects. *p*-values were adjusted with the Benjamini and Hochberg method, with a corrected *p*-value of 0.05 as the cutoff to define significant differential expression.

### 2.6. Target Gene Prediction and Functional Analyses

Predicted miRNA target genes were identified using a dual-algorithm approach: PITA (v6.0) and Miranda (v3.3a) were employed with stringent parameter settings to minimize false positives. For PITA, binding interactions were filtered by free energy change (ΔΔG ≤ −10 kcal/mol) and sequence complementarity (context score ≤ −0.1), while Miranda predictions required a seed region match score ≥ 160 and energy threshold ≤ −20 kcal/mol. Only targets predicted by both algorithms were retained for downstream analyses. Functional annotation of these target genes was performed through Gene Ontology (GO) enrichment analysis using the hypergeometric test (*p* ≤ 0.05, based on the January 2023 GO database), with terms categorized into biological processes, molecular functions, and cellular components [35]. Additionally, KOBAS 3.0 software was utilized to map targets to the KEGG PATHWAY database (2023.1 release), identifying significantly enriched pathways (*p* < 0.05) [36]. Significantly enriched KEGG pathways were identified via Fisher’s exact test.

### 2.7. miRNA Expression Profile Validation

To validate the accuracy of small sequencing results, the identical RNA samples used for library construction were subjected to qRT–PCR analysis. Specifically, cDNA synthesis was performed using a Mir-X miRNA First-Strand Synthesis Kit (TaKaRa, Dalian, China) according to the manufacturer’s protocol. Subsequent qRT–PCR validation was conducted on an FTC-3000 instrument (Funglyn Biotech, Toronto, Canada) with GoTaq^®^ qPCR Master Mix (2×) (Promega, Madison, WI, USA). Thermocycler settings were: 95 °C for 10 min; 40 cycles of 95 °C for 15 s, 60 °C for 15 s, and 95 °C for 15 s. Samples were analyzed in the form of three biological replicates and four technical replicates, while the U6 snRNA served as an internal control gene for these qRT–PCR analyses. Relative DEM expression was assessed via the 2^−ΔΔCt^ method [37]. Primer Premier 5.0 (http://www.premierbiosoft.com/primerdesign/index.html, accessed on 30 November 2023) was used for primer design (Table S1).

## 3. Results

### 3.1. Small RNA-Seq Data Analyses

To characterize the miRNA responses of adult *P. aenescens* to 20E stimulation, six sequencing libraries were prepared from adults two days following 20E injection. The raw sequencing data were deposited in the NCBI Short Read Archive (SRP) under BioProject ID PRJNA1166420. In total, these efforts yielded 154.87 million raw reads, with 40.61 to 12.83 million reads per library (Table S2). Following the removal of 5′ and 3′ adapter sequences, reads of low quality, and RNAs that were noted in the 18–25 nucleotide size range (ACGT101-miR), 35.46 million reads remained (Table S2), with 0.49 to 1.68 million unique small RNAs per library (Table S3). The length distributions of these small RNAs were bimodal, with peaks from 21–22 nt (Table S3). In total, 205 miRNAs were identified across these six combined libraries, among which 162 and 43 were, respectively, known and novel miRNAs (Table S4).

### 3.2. DEM Identificaiton

**Figure 1:**
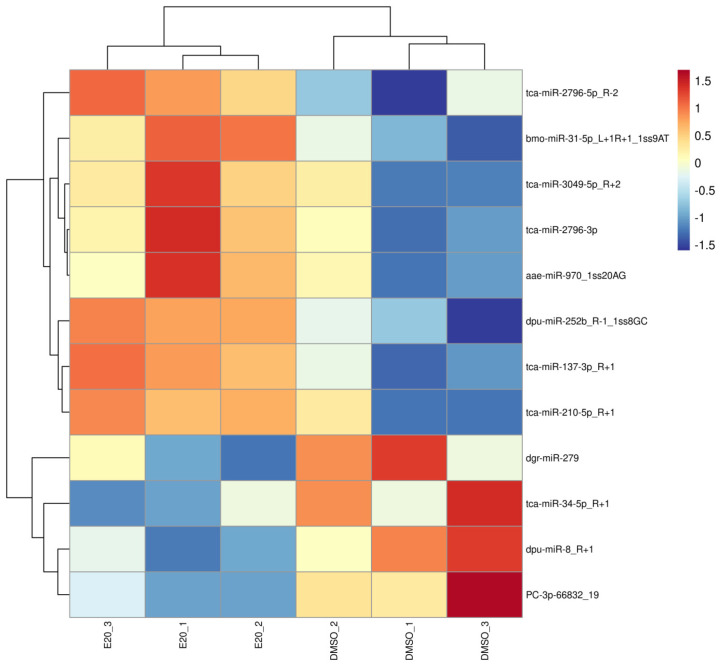
Heatmap of the miRNAs differentially expressed following treatment with 20E. Replicates are shown in columns, with colors representing expression levels from low (blue) to high (red).

Following treatment with 20E, 12 miRNAs were differentially expressed (eight upregulated, four downregulated), with *p* ≤ 0.05 (Figure 1, Table S5). Of these 12, miR-31-5p, miR-137-3p, miR-2796-5p, miR-252b, miR-2796-3p, miR-970, miR-3049-5p, and miR-210-5p were upregulated, while miR-8, PC-3p-66832_19, miR-34-5p, and miR-279 were downregulated.

### 3.3. Prediction and Functional Analyses of DEM Targets

**Figure 2:**
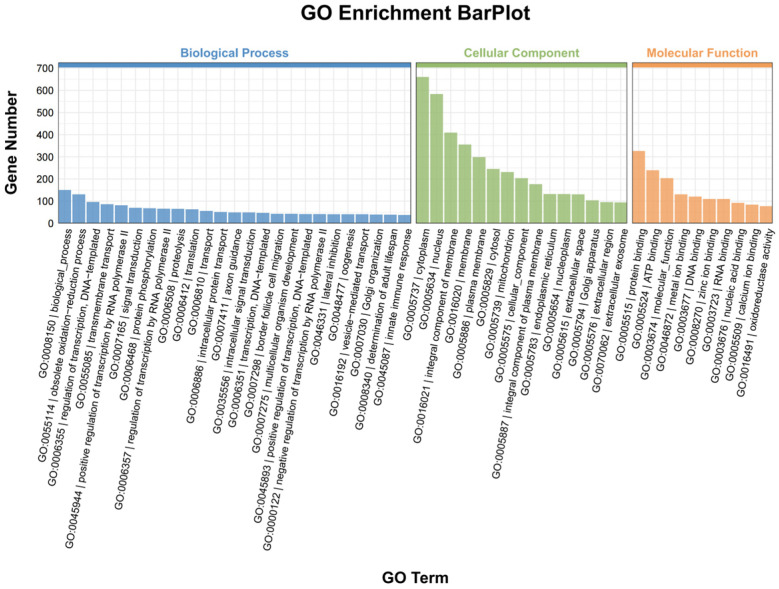
Histogram of GO enrichment results for the genes predicted to be targets of the miRNAs differentially expressed following treatment with 20E.

**Figure 3:**
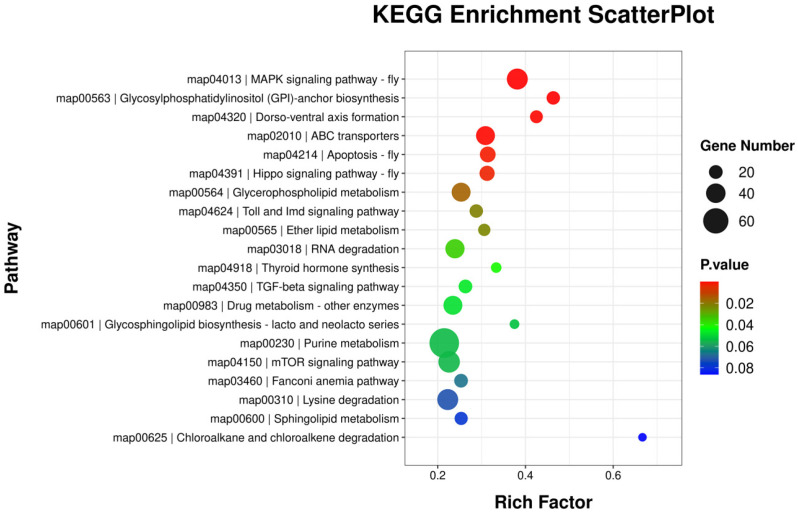
Scatter plot of KEGG enrichment results for the genes predicted to be targets of the miRNAs differentially expressed following treatment with 20E.

To gain insight into how DEMs function following 20E stimulation, their target genes were examined with the Miranda and TargetScan algorithms, yielding 7270 predicted target genes. The potential activities of these DEMs were then assessed through GO enrichment analyses of these putative target genes (Figure 2, Table S6). GO analyses revealed that 3616 predicted target genes were associated with 6557 GO terms, of which 319 were significantly enriched (*p* < 0.05). The most enriched biological process terms included ‘border follicle cell migration’, ‘cellularization’, and ‘Golgi organization’, whereas the most enriched molecular function terms included ‘protein phosphatase 1 binding’, ‘antiporter activity’, and ‘ATPase-coupled transmembrane transporter activity’, and the most enriched cellular component terms included ‘septate junction’, ‘apical part of cell’, and ‘presynaptic membrane’. Of these DEM target genes, 1733 were also associated with 329 KEGG pathways, among which 13 exhibited significant enrichment (*p* < 0.05). Of these pathways, the most significantly enriched were the ‘MAPK signaling pathway—fly’, ‘Glycosylphosphatidylinositol (GPI) anchor biosynthesis’, ‘Dorso–ventral axis formation’, ‘ABC transporters’, and ‘Apoptosis—fly’ pathways (Figure 3, Table S7).

### 3.4. Validation of Small RNA-Seq Results

**Figure 4:**
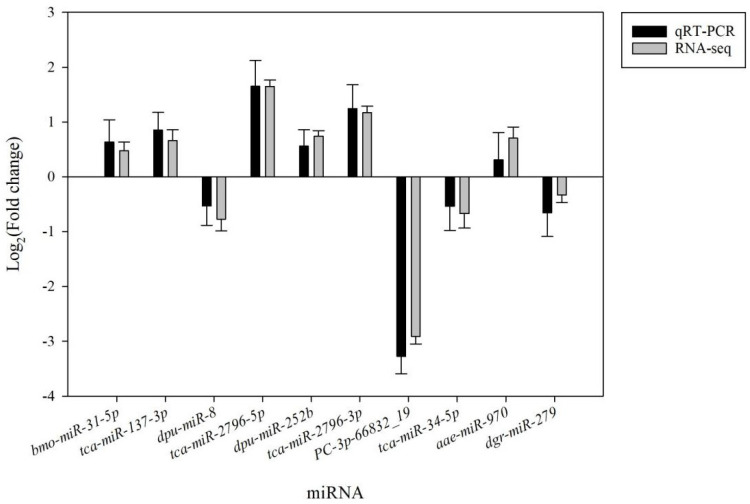
qRT–PCR-based validation of small RNA-Seq results for 10 miRNAs. Fold-change values include normalized small RNA-seq results and qRT–PCR values computed via the 2^−∆∆Ct^ method.

To confirm the validity of the above small RNA-seq analyses, 10 DEMs were selected at random and analyzed via qPCR (Figure 4). All 10 of these miRNAs exhibited changes in expression consistent with those detected via small RNA-seq, supporting the overall reliability of these small RNA-seq analyses.

## 4. Discussion

This study analyzed miRNA expression differences in adult *P. aenescens* following 48 h 20E treatment using sRNA-seq. The results showed that a total of 205 miRNAs were detected, with 12 significantly differentially expressed miRNAs (DEMs) identified. The predicted target genes of DEMs (3616 mRNAs) were significantly enriched in GO terms such as ‘border follicle cell migration’, ‘protein phosphatase 1 binding’, and ‘septate junction’ (Figure 3). KEGG pathway analysis revealed significant enrichment in key pathways including ‘MAPK signaling pathway—fly’, ‘Glycosylphosphatidylinositol (GPI) anchor biosynthesis’, ‘Dorso–ventral axis formation’, ‘ABC transporters’, and ‘Apoptosis—fly’ pathways’ (Figure 4). Further integration established the “20E–miRNA–*FOXO*/MAPK signaling pathway regulatory network”, elucidating the molecular mechanisms by which 20E governs insect metabolism and immunity through miRNA-mediated regulation. This study provides the first systematic analysis of 20E-responsive miRNA regulatory networks in *P. aenescens*, offering critical genetic resources to clarify hormone–miRNA interactions in insect physiology. These findings lay a theoretical foundation for developing miRNA-based targeted pest control strategies.

In past reports, interactions between a range of hormones and miRNAs have been shown to influence insect growth, development, and reproductive activity [38,39,40]. Hormones may exert their functions by interacting with both hormone signaling pathway-related genes and miRNA, the latter of which can also shape physiological processes through their effects on the hormone regulatory network [41,42]. In this study, the 20E treatment of adult *P. aenescens* for 48 h triggered the differential expression of 12 miRNAs, including eight that were upregulated and four that were downregulated. Notably, treatment with 20E resulted in the upregulation of miR-252b and miR-970, while downregulating miR-8 and miR-34-5p. Treatment with 20E similarly upregulated miR-970-3p in the silkworm embryonic cell line (BmE) and *Drosophila* S2 cell lines [22], and downregulated miR-34-5p in these cells [22]. However, even within the same hormone treatment, discrepancies exist: miR-8-5p was upregulated by 20E in the silkworm BmE cell line but was downregulated in *Drosophila* S2 cell lines [22], highlighting the species-specific nature of miRNA regulatory mechanisms. Moreover, 20E promoted miR-34-5p upregulation and miR-252b downregulation in *B.germanica* [19]; this contrasts sharply with the responses observed in *P. aenescens*. In the Coleoptera species *Galeruca daurica*, treatment with 20E for 48 h resulted in 52 differentially expressed miRNAs [24]; however, no common differentially expressed miRNAs were identified between *G. daurica* and *P. aenescens*. Such inconsistencies underscore the complexity of hormone–miRNA interactions. In conclusion, our results demonstrate that 20E-mediated miRNA modulation in *P. aenescens* shares both conserved and divergent features with other insects. These inconsistent results emphasize the species-specific differences in exogenous hormone treatment responses that can arise in insects, particularly with respect to miRNAs, supporting the complex interplay between the regulation of hormone signaling and miRNA-related pathways.

In this study, 20E treatment downregulated miR-8 in *P. aenescens*. Studies have demonstrated that 20E treatment leads to a significant downregulation of miR-8-3p, accompanied by a corresponding upregulation of its target gene trehalase (*SfTre1*), and subsequent experiments further validated that miR-8-3p modulates molting in *Sogatella furcifera* by directly targeting the *SfTre1* [43]. In other insects, miR-8 exhibits diverse developmental functions. For example, in *D. melanogaster*, miR-8 promotes corpus allatum (CA) cell growth and juvenile hormone (JH) biosynthesis [44], while in *Tribolium castaneum*, it regulates wing, eye, and leg morphogenesis by targeting developmental genes [45]. Collectively, these findings highlight a potential conserved mechanism by which 20E governs insect development through miR-8-mediated regulation of target genes. Further experimental validation is required to confirm this regulatory relationship and its functional implications in *P. aenescens*.

Upon binding to *EcR* and USP in a trimeric complex, 20E induces the expression of downstream primary transcription factors including *E93* and *Br-C* [46], which subsequently regulate secondary transcription factors that include *HR3* and *FTZ-F1*, initiating the transcriptional regulation of ecdysone-related genes to control the growth and development of insect species [47]. In *Nilaparvata lugens*, the 20E signaling axis has been shown to control ovarian development through the transcriptional control of genes including *E75*, *E74*, and *Br-C* [48,49]. Here, *Br-C* was identified as a miR-2796-5p and miR-137-3p target gene, and treatment with 20E significantly affected miR-2796-5p and *Br-C* expression. In *N. lugens*, 20E can negatively regulate miR-8-5p and miR-2a-3p through *Br-C*, thereby controlling the expression of *Tre-2* and *PAGM*, which are involved in chitin metabolism and nymph ecdysis, while the *Br-C*-mediated upregulation of miR-173 following 20E stimulation shapes nymphal molting through its ability to target *Ftz-f1* [50,51,52]. In *D. melanogaster*, 20E can stimulate the Let-7 Complex via *Br-C*, while 20E represses miR-34 [53]. Based on these results, miR-2796-5p may affect *P. aenescens* growth and development through the regulation of *Br-C* expression and activity in the ecdysone cascade.

KEGG enrichment analyses revealed 13 pathways that were significantly enriched, including several related to lipid metabolism as well as glycan biosynthesis and metabolism, suggesting that 20E may influence the expression of key genes associated with glycolipid metabolic processes in *P. aenescens*. *FoxO* is a highly conserved transcription factor that influences lipid metabolism [54], in addition to shaping a variety of physiological processes in insects that include growth, reproduction, longevity, and stress resistance. Prior studies have explored the relationship between 20E and *FoxO*, revealing that this hormone can upregulate *PTEN* expression and *Akt* phosphorylation, ultimately inhibiting *FoxO* phosphorylation and thereby activating this factor. As such, 20E can elicit the transcriptional activity of *FoxO*, whereupon it can shape intracellular transcriptional activity based on associated environmental and other signals [55]. The 20E-mediated enhancement of *FoxO* activity can drive the upregulation of brummer and acid lipase-l, inducing fat body lipid degradation in *B. mori* during the processes of molting and pupation [56]. 

The control of gene expression can take place at both the transcriptional and post-transcriptional levels [57], with miRNAs functioning as major post-transcriptional regulators in this context [58]. While many studies have explored the transcriptional activity of *FoxO* and how it regulates its target genes, the post-transcriptional miRNA-based regulation of *FoxO* is not understood. Few studies to date have been published on these regulatory interactions. In one such study, the disruption of pupal diapause was achieved in *Helicoverpa armigera* through the injection of a combination of ecdysone, diapause hormone, and diapause hormone analogs, which led to the downregulation of the insulin/*FoxO* signaling-related miR-277-3p [59]. Downregulating miR-277-3p has been shown to enhance nuclear *FoxO* export and to limit the storage of lipids in the fat body [60]. Here, four DEMs (miR-8, miR-2796-5p miR-31-5p, and miR-137-3p) were identified that were predicted to target 37 transcripts associated with the *FoxO* signaling pathway, including insulin-like receptor, epidermal growth factor receptor, serine/threonine-protein kinase PLK4 isoform X1, 5′-AMP-activated protein kinase subunit β-1, and phosphatidylinositol 3-kinase 60 (Table S8). These data support a potentially key role for these miRNAs in the regulation of lipid metabolic activity in *P. aenescens*.

The MAPK signal transduction pathway is a central mediator of the conversion of extracellular stimuli into signaling activity within recipient cells. MAPK signaling activity can regulate diverse physiological processes, including immunological defenses, stress responses, and metabolic activity, to influence appropriate homeostasis and preserve appropriate functionality under complex, dynamically changing conditions [61]. Here, the putative target genes of 20E-responsive DEMs were strongly enriched in the MAPK signaling pathway. In another Coleoptera species, *G. daurica*, significant enrichment of this pathway has similarly been reported [62]. Transcriptomic and proteomic analyses have similarly supported the significant enrichment of this pathway in the context of *Locusta migratoria* winter diapause as well as *Delia antique* summer diapause [63,64]. These findings suggest the ability of 20E to play a central role in the control of the growth and development of insects through miRNA-mediated effects on MAPK signaling activity.

This study is the first to characterize 20E-responsive miRNAs in *P. aenescens*, uncovering miR-2796-5p and miR-137-3p as potential regulators of *Br-C* in the ecdysone pathway. These findings provide novel molecular targets for disrupting pest reproduction. Identifying miRNAs (e.g., miR-8, miR-2796-5p) involved in lipid metabolism and *FoxO*/MAPK pathways offers opportunities for RNAi-based interventions. Targeting these miRNAs could disrupt 20E signaling, reducing fecundity and population growth. Although this study offers valuable insights into the miRNA regulatory networks in *P. aenescens* in response to 20E treatment, it has several limitations. The predicted miRNA–target gene interactions lack experimental validation, and in vivo functional studies are needed to confirm the proposed regulatory relationships. Future studies should validate miRNA–target interactions (e.g., miR-2796-5p/*Br-C*) via luciferase assays and explore miRNA-mediated lipid metabolism in vivo. Also, this study focused on whole-body samples, neglecting tissue-specific miRNA expression, and lacks integration with other omics data, which is necessary for a comprehensive understanding of the regulatory mechanisms.

## 5. Conclusions

This study aimed to systematically identify 20E-responsive miRNAs in *P. aenescens* and characterize their roles in reproductive and metabolic pathways. In total, 205 miRNAs were identified in this study, of which 162 were previously documented and 43 were novel. Relative to control (DMSO)-treated insects, 20E treatment resulted in the differential expression of 12 miRNAs (four downregulated, eight upregulated). Target prediction efforts suggested that these miRNAs may play a role in shaping the 20E-mediated regulation of *P. aenescens* growth and development via the *FoxO* and MAPK signaling pathways. These results provide new insight into the mechanisms governing 20E-related signal transduction in *P. aenescens* and the developmental effects of these processes, providing a strong foundation for future research focused on hormone signaling and the growth and reproductive development of other species of Coleoptera insects.

## Data Availability

The original contributions presented in this study are included in the article/supplementary material. Further inquiries can be directed to the corresponding author.

## Supplementary Materials

The following supporting information can be downloaded at: https://www.mdpi.com/article/10.3390/genes16040435/s1, Table S1: Primers used for qRT–PCR validation; Table S2: Summary of small RNA sequences; Table S3: The length distribution and abundance of miRNAs; Table S4: Summary of conserved and novel miRNAs; Table S5: Differentially expressed miRNAs; Table S6: GO enrichment analysis; Table S7: KEGG enrichment analysis; Table S8: DEMs and their target genes involved in the *FOXO* signaling pathway.

## References

[1] Feng W.Q., Wang S.J., Zhao J.Q., Duan J.P. (2015). Observation of biological characteristics and prevention methods in *Pyrrhalta aenescens*. J. Inner Mong. For..

[2] Wang S.J., Si Q., Zhang L.Y., Hua P.C., Liu Y.L., Li Y.Y. (2023). Laboratory toxicity and field efficacy of four insecticides against *Pyrrhalta aenescens* larva in Hohhot. Plant Prot..

[3] Humujiletu L., Xi W., Li N., Wu X.H., Wei C.G., Zhang Y. (2019). Research progress on characteristics and biological control of two elm leaf beetles. J. Inner Mong. For. Sci. Technol..

[4] Shen T.T., Shen T. (2025). Integrated control techniques of *Pyrrhalta aenescens*. Modern Horti..

[5] Wang P., Wang S.J., Tian X. (2022). Plant Diseases and Pests in Hohhot Gardens.

[6] Zhang B., Zhang W., Nie R.E., Li W.Z., Segraves K.A., Yang X.K., Xue H.J. (2016). Comparative transcriptome analysis of chemosensory genes in two sister leaf beetles provides insights into chemosensory speciation. Insect Biochem. Mol. Biol..

[7] Yao T.P., Forman B.M., Jiang Z., Cherbas L., Chen J.D., McKeown M., Cherbas P., Evans R.M. (1993). Functional ecdysone receptor is the product of EcR and ultraspiracle genes. Nature.

[8] Yamanaka N., Rewitz K.F., O’Connor M.B. (2013). Ecdysone control of developmental transitions: Lessons from drosophila research. Annu. Rev. Entomol..

[9] Yamanaka N. (2021). Ecdysteroid signalling in insects—From biosynthesis to gene expression regulation. Adv. Insect Physiol..

[10] Cáceres L., Necakov A.S., Schwartz C., Kimber S., Roberts I.J.H., Krause H.M. (2011). Nitric oxide coordinates metabolism, growth, and development via the nuclear receptor E75. Genes Dev..

[11] Kayukawa T., Nagamine K., Ito Y., Nishita Y., Ishikawa Y., Shinoda T. (2016). Krüppel Homolog 1 Inhibits Insect Metamorphosis via Direct Transcriptional Repression of Broad-Complex, a Pupal Specifier Gene. J. Biol. Chem..

[12] Pierceall W.E., Li C., Biran A., Miura K., Raikhel A.S., Segraves W.A. (1999). E75 expression in mosquito ovary and fat body suggests reiterative use of ecdysone-regulated hierarchies in development and reproduction. Mol. Cell Endocrinol..

[13] Beachum A.N., Whitehead K.M., McDonald S.I., Phipps D.N., Berghout H.E., Ables E.T. (2021). Orphan nuclear receptor ftz-f1 (NR5A3) promotes egg chamber survival in the *Drosophila* ovary. G3 Bethesda.

[14] Hossain M.S., Liu Y., Zhou S., Li K., Tian L., Li S. (2013). 20-Hydroxyecdysone-induced transcriptional activity of *FoxO* upregulates *brummer* and acid *lipase-1* and promotes lipolysis in *Bombyx* fat body. Insect Biochem. Mol. Biol..

[15] Ji C.H., Zhang N., Jiang H., Meng X.K., Ge H.C., Yang X.M., Xu X., Qian K., Park Y., Zheng Y. (2021). 20-hydroxyecdysone regulates expression of methioninesulfoxide reductases through transcription factor *FOXO* in the red flour beetle, *Tribolium castaneum*. Insect Biochem. Mol. Biol..

[16] Huang S.H., Yang H.H., Chen X.X., Zhang J.Y., Huang L.Q. (2018). Genomic transcriptional response to 20-hydroxyecdysone in the fat body of silkworm, *Bombyx mori*. Gene Rep..

[17] Bartel D.P. (2009). MicroRNAs: Target recognition and regulatory functions. Cell.

[18] Carthew R.W., Sontheimer E.J. (2009). Origins and mechanisms of miRNAs and siRNAs. Cell.

[19] Rubio M., de Horna A., Belles X. (2012). MicroRNAs in metamorphic and nonmetamorphic transitions in hemimetabolan insect metamorphosis. BMC Genom..

[20] Jin H., Kim V.N., Hyun S. (2012). Conserved microRNA miR-8 controls body size in response to steroid signaling in drosophila. Genes Dev..

[21] He K., Xiao H.M., Sun Y., Situ G., Xi Y., Li F. (2019). microRNA-14 as an efficient suppressor to switch off ecdysone production after ecdysis in insects. RNA Biol..

[22] Jin X.L., Wu X.Y., Zhou L.T., He T., Yin Q., Liu S.P. (2020). 20-hydroxyecdysone-responsive microRNAs of insects. RNA Biol..

[23] Guo S., Tian Z., Wu Q.W., King-Jones K., Liu W., Zhu F., Wang X.P. (2021). Steroid hormone ecdysone deficiency stimulates preparation for photoperiodic reproductive diapause. PLoS Genet..

[24] Wang H.C., Han H.B., Duan T.F., Li L., Pang B.P. (2022). Transcriptome-wide identification of microRNAs in response to 20-hydroxyecdysone in *Galeruca daurica*. Comp. Biochem. Physiol. Part D Genom. Proteom..

[25] Liu Z., Xu J., Ling L., Luo X., Yang D., Yang X., Zhang X., Huang Y. (2020). miR-34 regulates larval growth and wing morphogenesis by directly modulating ecdysone signalling and cuticle protein in *Bombyx mori*. RNA Biol..

[26] Wang Y., Bai Y.Y., Liu Y.X., Noel S.W., Yan Q.S., Thi H.P., Sun X.G., Wei W., Ma J., Zheng F. (2020). Plasma exosomal miRNAs involved in endothelial injury in microscopic polyangiitis patients. FASEB J..

[27] Peng Y., Jin M., Li H., Zhang L., Zhang L., Yu S. (2023). Population genomics provide insights into the evolution and adaptation of the Asia corn borer. Mol. Biol. Evol..

[28] Kozomara A., Griffiths-Jones S. (2011). miRBase: Integrating microRNA annotation and deep-sequencing data. Nucleic Acids Res..

[29] Langmead B. (2010). Aligning short sequencing reads with Bowtie. Curr. Protoc. Bioinform..

[30] Hofacker L.L., Bernhart S.H.F., Stadler P.F. (2004). Alignment of RNA base pairing probability matrices. Bioinformatics.

[31] Ambady S., Wu Z.Y., Dominko T. (2012). Identification of novel microRNAs in Xenopus laevis metaphase II arrested eggs. Genesis.

[32] Li X., Shahid M.Q., Wu J.W., Wang L., Liu X.D., Lu Y.G. (2016). Comparative small RNA analysis of pollen development in autotetraploid and diploid rice. Int. J. Mol. Sci..

[33] Zhou L., Chen J.H., Li Z.Z., Li X.X., Hu X.D., Huang Y., Zhao X.K., Liang C.Z., Wang Y., Sun L. (2010). Integrated profiling of microRNAs and mRNAs: MicroRNAs located on Xq27.3 associate with clear cell renal cell carcinoma. PLoS ONE.

[34] Anders S., Huber W. (2010). Differential expression analysis for sequence count data. Genome Biol..

[35] Young M.D., Wakefield M.J., Smyth G.K., Oshlack A. Goseq: Gene Ontology Testing for RNA-seq Datasets. https://bioconductor.org/packages//2.13/bioc/vignettes/goseq/inst/doc/goseq.pdf.

[36] Mao X.Z., Cai T., Olyarchuk J.G., Wei L.P. (2005). Automated genome annotation and pathway identification using the KEGG orthology (KO) as a controlled vocabulary. Bioinformatics.

[37] Livak K.J., Schmittgen T.D. (2001). Analysis of relative gene expression data using realtime quantitative PCR and the 2^−∆∆Ct^ method. Methods.

[38] Bryant B., Macdonald W., Raikhel A.S. (2010). microRNA miR-275 is indispensable for blood digestion and egg development in the mosquito *Aedes aegypti*. Proc. Natl. Acad. Sci. USA.

[39] Boulan L., Martin D., Milan M. (2013). Bantam miRNA promotes systemic growth by connecting insulin signaling and ecdysone production. Curr. Biol..

[40] Lozano J., Montanez R., Belles X. (2015). MiR-2 family regulates insect metamorphosis by controlling the juvenile hormone signaling pathway. Proc. Natl. Acad. Sci. USA.

[41] Herranz H., Cohen S.M. (2010). MicroRNAs and gene regulatory networks: Managing the impact of noise in biological systems. Genes Dev..

[42] Carthew R.W., Agbu P., Giri R. (2017). MicroRNA function in *Drosophila melanogaster*. Semin. Cell Dev. Biol..

[43] Ren Q.Q., Long G.Y., Yang H., Zhou C., Yang X.B., Yan Y., Yan X. (2024). Conserved microRNAs miR-8-3p and miR-2a-3 targeting chitin biosynthesis to regulate the molting process of *Sogatella furcifera* (Horváth) (Hemiptera: Delphacidae). J. Econ. Entomol..

[44] Zhang J., Wen D., Li E.Y., Palli S.R., Li S., Wang J., Liu S.N. (2021). MicroRNA miR-8 promotes cell growth of corpus allatum and juvenile hormone biosynthesis independent of insulin/IGF signaling in *Drosophila melanogaster*. Insect Biochem. Mol. Biol..

[45] Wu W., Zhai M., Li C., Yu X., Song X., Gao S., Li B. (2019). Multiple functions of miR-8-3p in the development and metamorphosis of the red flour beetle, *Tribolium castaneum*. Insect Mol. Biol..

[46] Ekoka E., Maharaj S., Nardini L., Dahan-Moss Y., Koekemoer L.L. (2021). 20-Hydroxyecdysone (20E) signaling as a promising target for the chemical control of malaria vectors. Parasites Vectors.

[47] Yoo B., Kim H.Y., Chen X., Shen W.P., Jang J.S., Stein S.N., Cormier O., Pereira L., Shih C.R.Y., Krieger C. (2021). 20-hydroxyecdysone (20E) signaling regulates amnioserosa morphogenesis during *Drosophila* dorsal closure: EcR modulates gene expression in a complex with the AP-1 subunit, Jun. Biol. Open.

[48] Carney G.E., Bender M. (2000). The *Drosophila* ecdysone receptor (EcR) gene is required maternally for normal oogenesis. Genetics.

[49] Ables E.T., Drummond-Barbosa D. (2010). The steroid hormone ecdysone functions with intrinsic chromatin remodeling factors to control female germline stem cells in *Drosophila*. Cell Stem Cell.

[50] Chen J., Liang Z.K., Liang Y.K., Pang R., Zhang W.Q. (2013). Conserved micrornas mir-8-5p and mir-2a-3p modulate chitin biosynthesis in response to 20-hydroxyecdysone signaling in the brown planthopper, *Nilaparvata lugens*. Insect Biochem. Mol. Biol..

[51] Chen J., Li T.C., Pang R., Yue X.Z., Hu J., Zhang W.Q. (2018). Genome-wide screening and functional analysis reveal that the specifc microRNA nlu-miR-173 regulates molting by targeting Ftz-F1 in *Nilaparvata lugens*. Front. Physiol..

[52] Song J.S., Zhou S.T. (2020). Post-transcriptional regulation of insect metamorphosis and oogenesis. Cell Mol. Life Sci..

[53] Sempere L.F., Sokol N.S., Dubrovsky E.B., Berger E.M., Ambros V. (2003). Temporal regulation of microRNA expression in *Drosophila melanogaster* mediated by hormonal signals and broad-Complex gene activity. Dev. Biol..

[54] Wei Q., Su J.Y. (2016). Research advances in carbohydrate and lipid metabolism in insects. Acta Entomol. Sin..

[55] Dong Y., Chen W.W., Kang K., Pang R., Dong Y.P., Liu K., Zhang W.Q. (2021). *FoxO* directly regulates the expression of *TOR/S6K* and *vitellogenin* to modulate the fecundity of the brown planthopper. Sci. China Life Sci..

[56] Hussain M., Frentiu F.D., Moreira L.A., O’Neill S.L., Asgari S. (2011). Wolbachia uses host microRNAs to manipulate host gene expression and facilitate colonization of the dengue vector *Aedes aegypti*. Proc. Natl. Acad. Sci. USA.

[57] Wei Y.Y., Chen S., Yang P.C., Ma Z.Y., Kang L. (2009). Characterization and comparative profiling of the small RNA transcriptomes in two phases of locust. Genome Biol..

[58] Asgari S. (2013). MicroRNA functions in insects. Insect Biochem. Mol. Biol..

[59] Reynolds J.A., Nachman R.J., Denlinger D.L. (2019). Distinct microRNA and mRNA responses elicited by ecdysone, diapause hormone and a diapause hormone analog at diapause termination in pupae of the corn earworm, Helicoverpa zea. Gen. Comp. Endocrinol..

[60] Ling L., Kokoza V.A., Zhang C.Y., Aksoy E., Raikhel A.S. (2017). MicroRNA-277 targets *insulin-like peptides 7* and *8* to control lipid metabolism and reproduction in *Aedes aegypti* mosquitoes. Proc. Natl. Acad. Sci. USA.

[61] Asl E.R., Amini M., Najafi S., Mansoori B., Mokhtarzadeh A., Mohammadi A., Lotfinejad P., Bagheri M., Shirjang S., Lotfi Z. (2021). Interplay between MAPK/ERK signaling pathway and microRNAs: A crucial mechanism regulating cancer cell metabolism and tumor progression. Life Sci..

[62] Duan T.F., Li L., Tan Y., Li Y.Y., Pang B.P. (2021). Identification and functional analysis of microRNAs in the regulation of summer diapause in *Galeruca daurica*. Comp. Biochem. Physiol. Part D Genom. Proteom..

[63] Hao Y.J., Zhang Y.J., Si F.L., Fu D.Y., He Z.B., Chen B. (2016). Insight into the possible mechanism of the summer diapause of *Delia antiqua* (Diptera: Anthomyiidae) through digital gene expression analysis. Insect Sci..

[64] Tu X.B., Wang J., Hao K., Whitman D.W., Fan Y.L., Cao G.C., Zhang Z.H. (2015). Transcriptomic and proteomic analysis of pre-diapause and nondiapause eggs of migratory locust, *Locusta migratoria L*. (Orthoptera: Acridoidea). Sci. Rep..

