# Supplementary material for: Transcriptomic Characterization of miRNAs in Pyrrhalta aenescens Fairmaire in Response to 20-Hydroxyecdysone Treatment
Source: Genes (Basel). 2025 Apr 5;16(4):435. doi: 10.3390/genes16040435 (PMC12026910; doi:10.3390/genes16040435)
Supplement: Supplementary file 1 [file genes-16-00435-s001.zip › Table S1 Primers used for qRT-PCR validation.pdf]

Table S1 Primers used for qRT-PCR validation

miRNA	Primer sequence(5'→ 3')
bmo-miR-31-5p	F:AGGCAAGATGTCGGCATAGCTGT
tca-miR-137-3p	F:TTATTGCTTGAGAATACACGTAG
dpu-miR-8	F:TAATACTGTCAGGTAAAGATGTCT
tca-miR-2796-5p	F:AGGGGTTTCTTTCGGCCTCCAG
dpu-miR-252b	F:CTAAGTACTAGTGCCGCAGGT
tca-miR-2796-3p	F:GTAGGCCGGCGGAAACTACTTGC
PC-3p-66832	F:AACTGCGAGTCTGTAGCGCCGT
tca-miR-34-5p	F:TGGCAGTGTGGTTAGCTGGTT
aae-miR-970	F:TCATAAGACACACGCGGCTGT
dgr-miR-279	F:TGACTAGATCCACACTCATTAA
U6	F: GGAACGATACAGAGAAGATTAGC
	R: TGGAACGCTTCACGAATTTGCG
	R: mRQ 3' primer
