# Supplementary material for: Transcriptomic Characterization of miRNAs in Pyrrhalta aenescens Fairmaire in Response to 20-Hydroxyecdysone Treatment
Source: Genes (Basel). 2025 Apr 5;16(4):435. doi: 10.3390/genes16040435 (PMC12026910; doi:10.3390/genes16040435)
Supplement: Supplementary file 1 [file genes-16-00435-s001.zip › Table S2 Summary of small RNA sequences.pdf]

Table S2 Summary of small RNA sequences in the libraries of *P. aenescens* adults

**Item**	**DMSO$_1$**		**DMSO$_2$**		**DMSO$_3$**	
	**Total (%)**	**Unique (%)**	**Total (%)**	**Unique (%)**	**Total (%)**	**Unique (%)**
Raw reads	13697612(100)	2906646(100)	60063968(100)	9967031(100)	13093535(100)	2952668(100)
3ADT & length filter	9546459(69.69)	2264487(77.91)	47440094(78.98)	8249115(82.76)	9436666(72.07)	2365320(80.11)
Junk reads	13863(0.10)	7482(0.26)	93952(0.16)	22489(0.23)	17508(0.13)	7288(0.25)
Clean reads	4137290(30.20)	2257005(77.65)	12529922(20.86)	8226626(82.54)	3639361(27.80)	2358032(79.86)
Rfam	387261(2.83)	8751(0.30)	683689(1.14)	11192(0.11)	262764(2.01)	7764(0.26)
Repeats	17323(0.13)	156(0.01)	78715(0.13)	315(0.00)	19236(0.15)	175(0.01)
Valid reads	3735785(27.27)	625840(21.53)	11779711(19.61)	1684056(16.90)	3360398(25.66)	572197(19.38)
rRNA	280273(2.05)	6530(0.22)	408640(0.68)	7163(0.07)	180597(1.38)	5633(0.19)
tRNA	46625(0.34)	946(0.03)	182839(0.30)	2208(0.02)	51265(0.39)	1079(0.04)
snoRNA	787(0.01)	50(0.00)	3290(0.01)	99(0.00)	926(0.01)	49(0.00)
snRNA	6119(0.04)	201(0.01)	15263(0.03)	454(0.00)	4945(0.04)	156(0.01)
other Rfam RNA	53457(0.39)	1024(0.04)	73657(0.12)	1268(0.01)	25031(0.19)	847(0.03)
**Item**	**E20$_1$**		**E20$_2$**		**E20$_3$**	
	**Total (%)**	**Unique (%)**	**Total (%)**	**Unique (%)**	**Total (%)**	**Unique (%)**
Raw reads	14570744(100)	2832881(100)	12829867(100)	2619441(100)	40612322(100)	6938009(100)
3ADT & length filter	9790299(67.19)	2274677(80.30)	8586576(66.93)	2119049(80.90)	31868925(78.47)	5831610(84.05)
Junk reads	18908(0.13)	6920(0.24)	12692(0.10)	5433(0.21)	33280(0.08)	12866(0.19)
Clean reads	4761537(32.68)	2267757(80.05)	4230599(32.97)	2113616(80.69)	8710117(21.45)	5818744(83.87)
Rfam	327345(2.25)	8063(0.28)	288382(2.25)	7594(0.29)	422589(1.04)	8992(0.13)
Repeats	26103(0.18)	224(0.01)	15427(0.12)	130(0.00)	46695(0.11)	216(0.00)
Valid reads	4412982(30.29)	543096(19.17)	3928550(30.62)	487286(18.60)	8246437(20.31)	1084418(15.63)
rRNA	225277(1.55)	5612(0.20)	217964(1.70)	5740(0.22)	272478(0.67)	6286(0.09)
tRNA	62494(0.43)	1253(0.04)	34893(0.27)	805(0.03)	81367(0.20)	1325(0.02)
snoRNA	1283(0.01)	68(0.00)	927(0.01)	54(0.00)	2027(0.00)	78(0.00)
snRNA	5747(0.04)	206(0.01)	4505(0.04)	149(0.01)	16704(0.04)	309(0.00)
other Rfam RNA	32544(0.22)	924(0.03)	30093(0.23)	846(0.03)	50013(0.12)	994(0.01)

3ADT&length filter: reads removed due to 3ADT not found and length with <18 and >26 were removed.

Junk reads:Junk: >=2N, >=7A, >=8C, >=6G, >=7T, >=10Dimer, >=6Trimer, or >=5Tetramer.

Clean reads: equal to raw reads - 3ADT&length filter - Junk reads.

Rfam:Collection of many common non-coding RNA families except micro RNA; http://rfam.janelia.org.

Repeats:Prototypic sequences representing repetitive DNA from different eukaryotic species; http://www.girinst.org/repbase.
