# Supplementary material for: Transcriptomic Characterization of miRNAs in Pyrrhalta aenescens Fairmaire in Response to 20-Hydroxyecdysone Treatment
Source: Genes (Basel). 2025 Apr 5;16(4):435. doi: 10.3390/genes16040435 (PMC12026910; doi:10.3390/genes16040435)
Supplement: Supplementary file 1 [file genes-16-00435-s001.zip › Table S3 The length distribution and abundance.pdf]

Table S3. The length distribution and abundance of small RNAs in the libraries of *P. aenescens* adults

Length	DMSO1		DMSO2		DMSO3	
	**Total (%)**	**Unique (%)**	**Total (%)**	**Unique (%)**	**Total (%)**	**Unique (%)**
18	205049(5.49)	29829(4.77)	464953(3.95)	67114(3.99)	153286(4.56)	22223(3.88)
19	244673(6.55)	39559(6.32)	545645(4.63)	91693(5.44)	177508(5.28)	30429(5.32)
20	344418(9.22)	53388(8.53)	866133(7.35)	129137(7.67)	265423(7.90)	44194(7.72)
21	636562(17.04)	131653(21.04)	2189180(18.58)	**313602(18.62)**	596080(17.74)	121100(21.16)
22	**855832(22.91)**	**97863(15.64)**	**3375035(28.65)**	245343(14.57)	**841745(25.05)**	**89518(15.64)**
23	584929(15.66)	71173(11.37)	1666658(14.15)	201271(11.95)	538695(16.03)	65374(11.43)
24	434369(11.63)	81009(12.94)	1311383(11.13)	248634(14.76)	421004(12.53)	77903(13.61)
25	429953(11.51)	121366(19.39)	1360724(11.55)	387262(23.00)	366657(10.91)	121456(21.23)
Valid reads	3735785(100)	625840(100)	11779711(100)	1684056(100)	3360398(100)	572197(100)
**Length**	**E201**		**E202**		**E203**	
	**Total (%)**	**Unique (%)**	**Total (%)**	**Unique (%)**	**Total (%)**	**Unique (%)**
18	257164(5.83)	30283(5.58)	167855(4.27)	21644(4.44)	335883(4.07)	43433(4.01)
19	261985(5.94)	35032(6.45)	194584(4.95)	27537(5.65)	342459(4.15)	56629(5.22)
20	341840(7.75)	43673(8.04)	293433(7.47)	37548(7.71)	534615(6.48)	77372(7.13)
21	738402(16.73)	**99948(18.40)**	652109(16.60)	**99579(20.44)**	1505421(18.26)	219566(20.25)
22	**1299329(29.44)**	77629(14.29)	**1135141(28.89)**	74972(15.39)	**2391994(29.01)**	160584(14.81)
23	680221(15.41)	62168(11.45)	677487(17.25)	57106(11.72)	1357639(16.46)	121928(11.24)
24	435861(9.88)	75541(13.91)	412471(10.50)	67223(13.80)	903769(10.96)	152498(14.06)
25	398180(9.02)	118822(21.88)	395470(10.07)	101677(20.87)	874657(10.61)	**252408(23.28)**
Valid reads	4412982(100)	543096(100)	3928550(100)	487286(100)	8246437(100)	1084418(100)
