# Supplementary material for: Transcriptomic Characterization of miRNAs in Pyrrhalta aenescens Fairmaire in Response to 20-Hydroxyecdysone Treatment
Source: Genes (Basel). 2025 Apr 5;16(4):435. doi: 10.3390/genes16040435 (PMC12026910; doi:10.3390/genes16040435)
Supplement: Supplementary file 1 [file genes-16-00435-s001.zip › Table S4 Summary of conserved and novel miRNAs.pdf]

Table S4 Summary of conserved and novel miRNAs in *P. aenescens*

No.	miRNA name	miRNA sequence	Length
1	tca-mir-3854-p5_1ss6AG	CCACAGTCCCAGCTCCACA	19
2	bmo-miR-1a-3p_R-1_1ss14AC	TGGAATGTAAAGACGTATGGA	21
3	bmo-miR-1a-3p_L-4	ATGTAAAGAAGTATGGAG	18
4	bmo-miR-1a-3p_R-2_1ss4AC	TGGCATGTAAAGAAGTATGG	20
5	bmo-miR-10-5p	ACCCTGTAGATCCGAATTTGT	21
6	bmo-miR-14-3p_R-1_1ss4GT	TCATTCTTTTTCTCTCTCCT	20
7	dps-mir-210a-p3_1ss11CT	GTACTTATTGTAGCTGCTG	19
8	bmo-miR-277-3p_R-4	TAAATGCACTATCTGGTAC	19
9	bmo-miR-277-3p_R-4	TAAATGCACTATCTGGTAC	19
10	bmo-miR-283-5p_L-1R+2_1ss9AC	AAATATCCGCTGGTAATTCTGG	22
11	bmo-miR-316-5p_R-4	TGTCTTTTTCCGCTTTGCT	19
12	bmo-miR-2779_L-1_1ss2TC	CATCCGGCTCGAAGGACCA	19
13	bmo-mir-2779-p3_1ss19AT	TCCGGCTCGAAGGACCATT	19
14	bmo-miR-2779_L-1_1ss2TC	CATCCGGCTCGAAGGACCA	19
15	bmo-mir-2779-p3_1ss18AG	CCGGCTCGAAGGACCATG	18
16	bmo-mir-2779-p5_1ss2TC	TCATATCCGGCTCGAAGGACCA	22
17	bmo-miR-2779_L-1_1ss2TG	GATCCGGCTCGAAGGACCA	19
18	bmo-miR-2779_1ss2TA	AAATCCGGCTCGAAGGACCA	20
19	mse-mir-2779-p3_1ss17AC	CCGGCTCGAAGGACCACT	18
20	bmo-mir-2779-p5_1ss20AT	ATCCGGCTCGAAGGACCATT	20
21	mse-miR-2779_L-1R+2	TCCGGCTCGAAGGACCACT	19
22	bmo-miR-2779_L-1	TATCCGGCTCGAAGGACCA	19
23	bmo-miR-2779_L-2_1ss20AG	ATCCGGCTCGAAGGACCG	18
24	mse-mir-2779-p5_1ss18AC	TCCGGCTCGAAGGACCACT	19
25	bmo-miR-2779_L-1_1ss2TA	AATCCGGCTCGAAGGACCA	19
26	bmo-miR-2779_L-2	ATCCGGCTCGAAGGACCA	18
27	bmo-mir-6497-p5_1ss9AG	AACTTCGGGATAAGGATTGGCTCT	24
28	bmo-miR-6497-5p_L-3_1ss11CT	CTGAGGATCGGGGCGTGTC	19
29	bmo-mir-6497-p5_1ss9AG	AACTTCGGGATAAGGATTGGCTCT	24
30	bmo-mir-6497-p3_1ss4AG	CGGGATAAGGATTGGCTCT	19
31	bmo-mir-6497-p5_1ss4AG	CGGGATAAGGATTGGCTCTGAGGA	24
32	bmo-mir-6497-p3_1ss18CT	AGGATTGGCTCTGAGGATCGGGGC	24
33	bmo-mir-6497-p5_1ss12AG	CGTAACTTCGGGATAAGGA	19
34	bmo-mir-6497-p3_1ss12AG	CGTAACTTCGGGATAAGG	18
35	isc-miR-1	TGGAATGTAAAGAAGTATGGAG	22
36	ame-miR-2-3p_L-2R-1	TCACAGCCAGCTTTGATGAG	20
37	tca-miR-7-5p_R-1	TGGAAGACTAGTGATTTTGTTGTT	24
38	tca-miR-7-3p_R-1	CAAGGAATCACTAATCATCCCA	22
39	tca-miR-7-5p_R-1	TGGAAGACTAGTGATTTTGTTGTT	24
40	tca-miR-7-3p_R-1	CAAGGAATCACTAATCATCCCA	22
41	bmo-miR-8-5p	CATCTTACCGGGCAGCATTAGA	22

42	dpu-miR-8_R+1	TAATACTGTCAGGTAAAGATGTCT	24
43	ame-miR-9a-5p	TCTTTGGTTATCTAGCTGTATGA	23
44	bmo-miR-9a-3p	ATAAAGCTAGGTTACCGGAGTTA	23
45	bmo-miR-9a-5p	TCTTTGGTTATCTAGCTGTATGA	23
46	bmo-miR-9a-3p	ATAAAGCTAGGTTACCGGAGTTA	23
47	der-miR-10_L+1	TACCCTGTAGATCCGAATTTGT	22
48	bmo-miR-10-3p	CAAATTCGGTTCTAGAGAGGTTT	23
49	dme-miR-10-5p_L+1R-1	TACCCTGTAGATCCGAATTTGT	22
50	dme-miR-10-3p	CAAATTCGGTTCTAGAGAGGTTT	23
51	tca-miR-11-3p	CATCACAGGCAGAGTTCTAGCT	22
52	dpu-miR-12	TGAGTATTACATCAGGTACTGGT	23
53	aga-miR-13b	TATCACAGCCATTTTGACGAGT	22
54	dqu-miR-13a-3p_1ss18TC	TATCACAGCCATTTTGACGAGC	22
55	api-miR-14	TCAGTCTTTTTCTCTCTCCTAT	22
56	ame-miR-31a-5p_L+1R-2	AGGCAAGATGTCGGCATAGCT	21
57	bmo-miR-31-5p_L+1R+1_1ss9AT	AGGCAAGATGTCGGCATAGCTGT	23
58	pca-miR-31-5p_R-2	AGGCAAGATGTCGGCATAGCT	21
59	tca-miR-34-5p_R+1	TGGCAGTGTGGTTAGCTGGTT	21
60	tca-miR-34-3p_R-2_1ss18CT	CGACCACTATCCATACTTCCT	21
61	tca-miR-71-5p	TGAAAGACATGGGTAGTGAGAT	22
62	tca-miR-71-3p	TCTCACTACCTTGTCTTTCATG	22
63	tca-miR-71-5p	TGAAAGACATGGGTAGTGAGAT	22
64	tca-miR-71-3p	TCTCACTACCTTGTCTTTCATG	22
65	tca-miR-87b-5p	ACGCTTGAACTTTGTTTTTCCT	22
66	tca-miR-87b-3p_R-2	GTGAGCAAAGATTCAGGTGTGT	22
67	aae-miR-92b-3p	AATTGCACTTGTCCCGGCCTGC	22
68	tca-miR-92c-3p_R-1	TATTGCACCAGTCCCGGCCTGA	22
69	dpu-miR-100_R-1	AACCCGTAGATCCGAACTTGTG	22
70	aae-miR-124_R+2	TAAGGCACGCGGTGAATGCCA	21
71	tcf-miR-125_1ss14AT	TCCCTGAGACCCTTACTTGTGA	22
72	dpe-miR-133	TTGGTCCCCTTCAACCAGCTGT	22
73	tca-miR-137-5p_R-2	ACGTGTATTCTTGGGTAATTAAC	23
74	tca-miR-137-3p_R+1	TTATTGCTTGAGAATACACGTAG	23
75	tca-miR-184-5p_1ss14TA	CCTTGTCATTCTCACGCCCGGT	22
76	tca-miR-184-3p_R+1	TGGACGGAGAACTGATAAGGGCT	23
77	tca-miR-190-5p_R+3	AGATATGTTTGATATTCTTGGTTG	24
78	tca-miR-190-3p_R+4	CCCAGGAGTCAAACATATTATT	22
79	tca-miR-193-3p_R-1	TACTGGCCTGTTAAGTCCCAAG	22
80	tca-miR-210-5p_R+1	AGCTGCTGGACGCTGCACAAGA	22
81	tca-miR-210-3p_L+1R-1	CTTGTGCGTGTGACAGCGGCT	21
82	aae-miR-219_R-2	TGATTGTCCAAACGCAATTCT	21
83	dme-miR-252-5p_R-1	CTAAGTACTAGTGCCGCAGGA	21
84	dme-miR-252-3p_L-2R+1_2ss10CT22TC	CTGCTGCTCAAGTGCTTATCA	21

85	dpu-miR-252b_R-1_1ss8GC	CTAAGTACTAGTGCCGCAGGT	21
86	bmo-miR-263a-5p_1ss10GA	AATGGCACTAGAAGAATTCAC	21
87	bmo-miR-263a-5p_1ss10GA	AATGGCACTAGAAGAATTCAC	21
88	dqu-miR-263b-5p_R-1	CTTGGCACTGGAAGAATTCACAG	23
89	aga-miR-275	TCAGGTACCTGAAGTAGCGCGCG	23
90	aga-miR-276-5p_R+1	AGCGAGGTATAGAGTTCCTAC	21
91	aga-miR-276-3p	TAGGAACTTCATACCGTGCTCT	22
92	aga-miR-276-5p_R+1	AGCGAGGTATAGAGTTCCTAC	21
93	aga-miR-276-3p	TAGGAACTTCATACCGTGCTCT	22
94	aae-miR-277-3p_R-1	TAAATGCACTATCTGGTACGA	21
95	dgr-miR-279	TGACTAGATCCACACTCATTAA	22
96	tca-miR-279d-3p_R-2	TGACTAGATCCATACTCGTCTAT	23
97	tca-miR-279e-3p_R-4_1ss10CG	TGACTAGATGGAACACTCGC	20
98	lmi-miR-281-5p	AAGAGAGCTATCCGTCGACAGT	22
99	lmi-miR-281-3p_L+1R-2	CTGTCATGGAGTTGCTCTCTTT	22
100	bmo-miR-281-5p	AAGAGAGCTATCCGTCGACAGT	22
101	bmo-miR-281-3p_L-1R+1	CTGTCATGGAGTTGCTCTCTTT	22
102	bmo-miR-281-5p	AAGAGAGCTATCCGTCGACAGT	22
103	bmo-miR-281-3p_L-1R+1	CTGTCATGGAGTTGCTCTCTTT	22
104	aae-miR-282-5p_L-4R-1	TAGCCTCTCCTAGGCTTTGTCT	22
105	ame-miR-283-5p_R+2	AAATATCAGCTGGTAATTCTGG	22
106	dan-miR-285_1ss21GT	TAGCACCATTCGAAATCAGTTC	22
107	tca-miR-305-5p_R+3	ATTGTACTTCATCAGGTGCTCTGG	24
108	tca-miR-305-3p_L+1R-1	CCGACACCTGTTGGAGTGCACT	22
109	bmo-miR-307-5p_R-1	ACTCACTCAACCTGGGTGTGAT	22
110	bmo-miR-307-3p_R+1	TCACAACCTCCTTGAGTGAGC	21
111	tca-miR-308-3p_R-1	AATCACAGGAAAATTCTGTGC	21
112	ame-miR-315-5p_R+1	TTTTGATTGTTGCTCAGAAAGCC	23
113	api-miR-316_R-3	TGTCTTTTTCCGCTTTGCTG	20
114	aae-miR-317	TGAACACAGCTGGTGGTATCT	21
11	tca-miR-375-3p_R-2_1ss17GA	TTTGTTCGTGTGGCTCAAGT	20

5			
116	dqu-miR-750-3p_R-5	CCAGATCTAACTCTTCCA	18
117	tca-miR-750-3p_R-5_1ss20AG	CCAGATCTAACTCTTCCATG	20
118	tca-miR-927a-5p_R+2	TTTAGAATTCCTACGCTTTACC	22
119	tca-miR-927a-3p_R-1	CAAAGCGTTTGGATTCTGAATC	22
120	tca-miR-927a-5p_R+2	TTTAGAATTCCTACGCTTTACC	22
121	tca-miR-927a-3p_R-1	CAAAGCGTTTGGATTCTGAATC	22
122	tca-miR-927b-5p_R+2	TTTAGAATCTGTACGCTTTGTTG	23
123	tca-miR-929-5p_L+1R-1	AAATTGACTCTAGTAGGGAGT	21
124	tca-miR-929-3p	CTCCCTAACGGAGTCAGGTTG	21
125	tca-miR-929-5p_L+1R-1	AAATTGACTCTAGTAGGGAGT	21
126	tca-miR-929-3p	CTCCCTAACGGAGTCAGGTTG	21
127	tca-miR-932-5p	TCAATTCCGTAGTGCATTGCAGT	23
128	tca-miR-932-3p_R+1	TGCAAGCAGTGCGGAAGTGAGG	22
129	dqu-miR-965-3p_R+1	TAAGCGTATAGCTTTTCCCCTT	22
130	aae-miR-970_1ss20AG	TCATAAGACACACGCGGCTGT	21
131	tca-miR-971b-5p	CACTCTAAGTTTGAACACCAAGC	23
132	tca-miR-971b-3p	TTGGTGTTCTACCTTACAGTGAG	23
133	pca-miR-980-3p_R+3	TAGCTGCCTTTTGAAGGGCAAT	22
134	hme-miR-981	TTCGTTGTCGACGAAACCTGCA	22
135	api-miR-981_R-2_1ss12TC	TTCGTTGTCGACGAAACCTT	20
136	tca-miR-989-3p_R+5	TGTGATGTGACGTAGTGGTATGT	23
137	bmo-miR-993a-5p_L-2R+1	TACCCTGTAGATCCGGGCTTTTG	23

13<br>8	ame-miR-993-3p	GAAGCTCGTCTCTACAGGTATCT	23
13<br>9	tca-miR-993-5p_R+3	TACCCTGTAGATCCGGGCTTTTG	23
14<br>0	tca-miR-993-3p	GAAGCTCGTCTCTACAGGTATCT	23
14<br>1	tca-miR-995-3p_R-2	TAGCACCACATGATTCAGCTTA	22
14<br>2	tca-miR-998-3p	TAGCACCATGGGATTCAGCTCA	22
14<br>3	api-miR-1000_R-1	ATATTGTCCTGTCACAGCAGT	21
14<br>4	dqu-miR-1175-5p_R-4_1ss20AT	AAGTGGAGAAGTGGTCTCTT	20
14<br>5	dqu-miR-1175-3p_1ss24TG	TGAGATTCAACTCCTCCAACTTAG	24
14<br>6	bmo-miR-1175-3p_1ss24AG	TGAGATTCAACTCCTCCAACTTAG	24
14<br>7	tca-miR-2765-5p_R+1	TGGTAACTCCACCACCGTTGGCG	23
14<br>8	tca-miR-2765-3p_L+1R-1	CCAGCGGTCGAGGAGTTCCTTAA	23
14<br>9	bmo-miR-2779_L+2R+2	TTATATCCGGCTCGAAGGACCACA	24
15<br>0	tca-miR-2788-5p	TGGGGTTTCTTAGCGGCATTT	21
15<br>1	tca-miR-2788-3p_R+3	CAATGCCCTTGGAAATCCCAAA	22
15<br>2	tca-miR-2796-5p_R-2	AGGGGTTTCTTTCGGCCTCCAG	22
15<br>3	tca-miR-2796-3p	GTAGGCCGGCGGAAACTACTTGC	23
15<br>4	tca-miR-2944c-5p_L-1_1ss10TC	AAGGAACTCCTGGTGTGATATG	22
15<br>5	tca-miR-2944c-3p_R+1	TATCACAGCCAGTAGTTACCT	21
15<br>6	aga-miR-2944b-5p_L-1_1ss12CT	AAGGAACTCCTGGTGTGATATG	22
15<br>7	tca-miR-3049-5p_R+2	TCGGGAAGACAGTTGCGGCGGATT	24
15<br>8	tca-miR-3477-5p_R-2	TAATCTCATTTGGTAACTGTGA	22
15<br>9	dqu-miR-3477-5p_1ss10GT	TAATCTCATTCGGTAACTGTGA	22
16	tca-miR-3849-5p_R+2	TGACATTTTAACCATAGTGCTGT	23

0			
161	tca-miR-6012-5p_L+3R+3	AGACTGATCTTGTTTCTGCGGAAAT	25
162	tca-miR-6012-3p_R-1_1ss10GA	TTCGGCGATAAGATCAGCCTGT	22
163	tca-let-7-5p_R+1	TGAGGTAGTAGGTTGTATAGT	21
164	tca-let-7-3p_R-1	CTGTACAGCCTGCTAACTTTCC	22
165	PC-3p-87456_15	TACTACAAATGTGACACGTAGAGGC	25
166	PC-3p-87456_15	TACTACAAATGTGACACGTAGAGGC	25
167	PC-5p-27755_112	CGCCGATACGGACTCGTAGTTC	22
168	PC-3p-75043_19	AACTGCGAGTCTGTAGCGCCGT	22
169	PC-5p-2761_1323	TTTGTCTATCATGATCATCTCT	22
170	PC-3p-10216_370	AGAGAGATGGTAATGATAGACT	22
171	PC-5p-19570_178	AGGGTTTTTCAAAAACTGTTGAA	23
172	PC-3p-41512_58	CACGGTTTTTGAAAAACTTTTA	22
173	PC-5p-79937_17	AAACGGATCAAGATATGT	18
174	PC-5p-79937_17	AAACGGATCAAGATATGT	18
175	PC-3p-134432_6	ATTCGTTGATTGTCCCACAGTTG	23
176	PC-5p-34369_81	TCGAGGAAGGGGAATGGGCACGATT	25
177	PC-3p-43289_54	GGGCAGCTTCCGGGAAAC	18
178	PC-5p-161121_5	GAAGAGGACGTGGTCGTG	18
179	PC-3p-135053_6	TACAAGTTGATGGAAGAGTGTATGT	25
180	PC-5p-85050_15	TACGAGACTACTGCTGCTAGAG	22
181	PC-5p-58804_31	ATATTTTTACATTTTTGGACT	21
182	PC-5p-23348_142	TGACTAGGTGATGTTGGGAAAGATA	25

18<br>3	PC-3p-95365_12	TACGCTTCGGGCTTGCACTCG	21
18<br>4	PC-5p-114241_9	AAAGAACGATCTTGAAGCT	19
18<br>5	PC-3p-65459_25	TGGAAGAATTCGAGAAGCT	19
18<br>6	PC-5p-79170_18	CTAAAGTTGGATAATTTTT	19
18<br>7	PC-5p-107957_10	TCCAATGGTGTGACATGAAT	20
18<br>8	PC-3p-102774_11	GTCACACGAACTTTACTTT	19
18<br>9	PC-3p-35621_76	TGAGAAATAATGTGGAAGAT	20
19<br>0	PC-3p-56533_33	TGGAAAAGTAGTTCGAAAAGTACA<br>C	25
19<br>1	PC-5p-84850_15	TTCTGACACGTTTATTACTCCACGG	25
19<br>2	PC-3p-166821_5	TACTATCCGAAGACGAAAAAC	21
19<br>3	PC-3p-51741_39	TATCATTATGAAACGGATTTTGGGC	25
19<br>4	PC-3p-112229_9	GTAAAAAATTGGCACTACGA	20
19<br>5	PC-3p-18136_195	AAACTTGATCATTTAGAGGA	20
19<br>6	PC-5p-7408_513	GAAGAAAGAATCCGAACCTGA	21
19<br>7	PC-5p-604_6130	TTTGTGAAAAAGATTTTGTAC	21
19<br>8	PC-5p-80359_17	TTTTACTTGATGCTAAGGTCCTCT	24
19<br>9	PC-3p-65706_25	TCAGACCGACGATGGACCCA	20
20<br>0	PC-5p-32541_88	AGCAGTTACAATGACCCTGGACCTT	25
20<br>1	PC-5p-108897_10	AATGATAATACTGTAAGAGT	20
20<br>2	PC-3p-90822_14	TACTATCTCGAATTCTCT	18
20<br>3	PC-3p-185785_5	TGTTGTCGGGGTCAAGCCATGAGCT	25
20<br>4	PC-5p-103941_11	TATGATACTGAACAATAAAACAAT	24
20	PC-5p-9202_412	CGAATTTGCCAATAAAAACTCGCTA	25

5			

The miR_name is composed of the 1st known miR name in a cluster, a underscore, and a matching annotation: such as L-n means the miRNA_seq (detected) is n base less than known rep_miRSeq in the left side; R-n means the miRNA_seq (detected) is n base less than known rep_miRSeq in the right side; L+n means the miRNA_seq (detected) is n base more than known rep_miRSeq in the left side; R+n means the miRNA_seq (detected) is n base more than known rep_miRSeq in the right side; 2ss5TC13TA means 2 substitution (ss), which are T->C at position 5 and T->A at position 13.

If there is no matching annotation, the miRNA_seq (detected) is exactly same as known rep_miRSeq.

New discovered 5p/3p sequence has been annotated as p3/p5: which is directly differentiate with the reported sequences,
